# Characterization of an endoplasmic reticulum stress‐related signature to evaluate immune features and predict prognosis in glioma

**DOI:** 10.1111/jcmm.16321

**Published:** 2021-02-21

**Authors:** Qing Zhang, Gefei Guan, Peng Cheng, Wen Cheng, Lianhe Yang, Anhua Wu

**Affiliations:** ^1^ Department of Neurosurgery The First Hospital of China Medical University Shenyang China; ^2^ Department of Pathology First Affiliated Hospital and College of Basic Medical Sciences of China Medical University Shenyang China

**Keywords:** endoplasmic reticulum stress, endoplasmic reticulum stress risk model, glioma, immunosuppressive, overall survival, tumour microenvironment

## Abstract

Endoplasmic reticulum (ER) stress has considerable impact on cell growth, proliferation, metastasis, invasion, angiogenesis and chemoradiotherapy resistance in various cancers. However, the effect of ER stress on the outcomes of glioma patients remains unclear. In this study, we established an ER stress risk model based on The Cancer Genome Atlas (TCGA) glioma data set to reflect immune characteristics and predict the prognosis of glioma patients. Survival analysis indicated that there were significant differences in the overall survival (OS) of glioma patients with different ER stress‐related risk scores. Moreover, the ER stress‐related risk signature, which was markedly associated with the clinicopathological properties of glioma patients, could serve as an independent prognostic indicator. Functional enrichment analysis revealed that the risk model correlated with immune and inflammation responses, as well as biosynthesis and degradation. In addition, the ER stress‐related risk model indicated an immunosuppressive microenvironment. In conclusion, we present an ER stress risk model that is an independent prognostic factor and indicates the general immune characteristics in the glioma microenvironment.

## 1 INTRODUCTION

Glioma is the most prevalent primary brain tumour in adults. As well as being highly invasive, it is characterized by diffuse infiltration, fuzzy boundaries and aggressive proliferation.^1^ With the development of molecular biological techniques, our understanding of the pathogenesis of glioma has greatly improved, and clinically, important genetic changes have been identified. In the revised classification of central nervous system (CNS) tumours proposed by the World Health Organization (WHO) in 2016, gliomas were classified based on a combination of histological findings and molecular findings, namely isocitrate dehydrogenase (IDH) mutations, 1p19q codeletion, H3 Lys27Met and RELA‐fusion.^2^ However, although the diagnosis and treatment of gliomas have been greatly improved, the outcome of glioma patients is still unfavourable, with a mortality rate of nearly 80% during the first year of diagnosis.^3^ For glioblastomas (GBMs), median survival is only approximately 15 months.^4^ Thus, there is an urgent need to improve the diagnosis and treatment of glioma.

As the largest organelle in eukaryotic cells, ER is a membrane structure composed of branched tubules and flat sacs, and is a major site of protein synthesis, processing and transport.^5^ However, the protein‐processing capacity of the ER is finite. When the protein folding capacity of the ER is exceeded, the cell is considered to be in a state of ER stress state.^6^ Many factors can reduce the efficiency of protein folding and lead to ER stress, including oxidative stress, nutrient deprivation, proteotoxicity, hypoxia and metabolic stress, as well as impaired calcium balance.^7^, ^8^ It is generally believed that ER stress is triggered by three branches of transmembrane ER sensors: IRE1α, PERK and ATF6. Misfolded proteins are continuously monitored by these three receptors, and when the concentration of misfolded proteins reach a certain level, the sensors trigger the ER stress response.^9^ Several studies have confirmed that chronic ER stress is a typical feature of many diseases, including tumours.^10^

In tumours, the high metabolism and proliferation of tumour cells lead to ischaemia and hypoxia within the tumour, causing tumour cells to enter a state of continuous ER stress. Signal transduction and regulation induced by the ER stress state promote tumour growth, angiogenesis, immune escape and chemoradiotherapy resistance.^11^, ^12^ ER stress can stimulate tumour cells to secrete a heat‐resistant factor, which can act on leucocytes around tumour cells, thereby changing the local immune characteristics of the tumour and promoting the growth of tumour cells.^13^ In addition, macrophages secrete VEGF in response to treatment with the conditioned medium of tumour cells under ER stress, thus enhancing angiogenesis within the tumour microenvironment.^14^

In gliomas, ER stress can change the metabolic state of tumour cells to promote tumorigenesis and therapy resistance.^15^, ^16^ Therefore, ER stress has become a new target for glioma therapy.^17^ General ER stress activators^18^ and the selective ATF6,^19^ PERK^20^ or GRP78^21^ activators have been confirmed to regulate multiplication and to facilitate apoptosis of glioma cells. However, some small molecules that interfere with protein processing and folding can inhibit the growth of tumour cells.^22^ Therefore, aberrant expression of ER stress‐related genes may have prognostic value for glioma patients and can be exploited as potential therapeutic targets.

In this study, we developed an ER stress‐related risk model, which can not only accurately predict the outcomes of glioma patients, but also distinguish the immune characteristics of glioma. In addition, we established a nomogram that integrates the prognosis model with clinicopathological factors (age, gender, grade, IDH mutation status, 1p19q codeletion status and MGMT promoter methylation status) and found its performance in estimating 1‐, 3‐ and 5‐year survival rates of glioma patients is excellent.

## 2 MATERIALS AND METHODS

### 2.1 Data sets and data collection

GeneCards (https://www.genecards.org/) is a searchable, integrative database that provides comprehensive, user‐friendly information on all annotated and predicted human genes. ER stress‐related genes were extracted from GeneCards, and genes with a relevance score ≥7 were selected. TCGA RNA‐seq transcriptome data and clinical information were obtained from TCGA database (http://cancergemome.nih.gov/). The Chinese Glioma Genome Atlas (CGGA) mRNA expression data (mRNAseq_325 and mRNA‐array) and corresponding clinicopathological features were collected from the CGGA database (http://www.cgga.org.cn). The GSE16011 data set was procured from the Gene Expression Omnibus (GEO) database (http://www.ncbi.nlm.nih.gov/geo/). Raw data were processed via the R package, *affy*, and robust multi‐array analysis (RMA) was used for background correction and normalization. Biospecimens and clinical data from related research were used as supplements.^23^ Patient characteristics, including detailed data for each research object, are shown in Table S1.

### 2.2 Samples and quantitative real‐time PCR analysis

From June 2018 to September 2019, we gathered 12 glioma tissues (4 cases each of grade II, III and IV glioma) from patients at the First Hospital of China Medical University. The clinical characteristics of 12 patients were shown in Table S2. Total RNA extraction and quantitative real‐time PCR (qRT‐PCR) was performed as previously described.^24^ After extraction using TRIzol reagent (Invitrogen/Thermo Fisher Scientific), total RNA was transcribed to first‐strand cDNA and then underwent qRT‐PCR (SYBR Green Master Mix). Each sample was assayed in triplicate. After being normalized to GAPDH expression levels, the expression values were log_2_ transformed. Primer sequences of target genes are shown in Table S3. This research was approved by the Medical Ethics Committee of the First Affiliated Hospital of China Medical University. All participants provided written informed consent.

### 2.3 Western blot assay

Western blot assay was performed as previously described.^24^ Briefly, the total protein of glioma tissues was extracted using a protein extraction kit (Beyotime). Equivalent amounts of protein (25‐50 μg) were then electrophoresed and transferred to polyvinylidene fluoride membranes (0.45 μm; Millipore). After being blocked, membranes were incubated with antibodies against ATF6 (24169‐1‐AP, Proteintech), EIF2α (sc‐133132, Santa), p‐EIF2α (# 3398S, CST), p‐IRE1α (sc‐390960, Santa) and GAPDH (60004‐1‐Ig, Proteintech) at 4°C for 16 hours. The membranes were then incubated with appropriate secondary antibodies (ProteinTech). Protein bands of interest were detected and quantified using a chemiluminescence kit (Beyotime), the ChemiDoc™ Touch detection system (Bio‐Rad Laboratories) and Image J software (National Institutes of Health).

### 2.4 ER stress‐related risk signature construction and validation

We first conducted univariate Cox regression and Kaplan‐Meier (KM) analysis using the *survival* R package to identify ER stress‐related genes associated with patients’ overall survival (OS) time from TCGA, CGGA (CGGA refers to mRNAseq_325 data unless otherwise specified), CGGA (mRNA‐array) and GSE16011 data sets. Only when the p values of both analysis methods were ≤0.05 were the genes included in the next step. The intersection genes related to OS from the above four data sets were analysed by least absolute shrinkage and selection operator (LASSO) regression, using the *glmnet* R package in TCGA database, to narrow the range of prognosis‐related genes. Subsequently, the Akaike information criterion (AIC) method of multivariate Cox regression analysis was performed using the *survival* package to establish an optimal ER stress‐related risk signature based on linear integration of the regression coefficient obtained from the multivariate Cox regression analysis and expression level of the selected ER related genes. The risk score was computed as follows:

$$Riskscore=\sum_{i=1}^{N}\left({\mathrm{Coef}}_{i},\times ,{\mathrm{Exp}}_{i}\right)$$

where *Exp_i_* is the expression value of the ER stress‐related genes and *Coef_i_* is the corresponding regression coefficient calculated by multivariate Cox regression analysis. TCGA data were used as the training cohort, and CGGA and GSE16011 data were used for the validation cohorts.

### 2.5 Survival analysis

Kaplan‐Meier survival analyses were performed using the *survival* and *survminer* packages in R to compare the OS between different groups of glioma patients. The R package *survivalROC* was used to establish time‐dependent receiver operating characteristic (ROC) curves to check the accuracy of the risk signatures in predicting the outcomes of glioma patients. The larger the area under the ROC curve (AUC), the stronger the predictive ability of the risk model. A risk plot was drawn using *Pheatmap* R package to display the distribution of survival status of samples in different risk groups.

### 2.6 Functional enrichment analysis

The *GSVA* package in R was applied to estimate the Gene Ontology (GO) biological processes and Kyoto Encyclopedia of Genes and Genomes (KEGG) pathways that correlated with the risk signature. The *GSVA* package scored the GO biological processes and KEGG pathways in each sample, and then by comparing the score differences in different risk groups, we identified the different biological processes enriched in the high‐ and low‐risk groups. The *limma* package in R was adopted to identify differentially expressed genes and gene sets in different groups. To further verify the GO processes and KEGG pathways related to the signature, GO and KEGG analyses were performed for the differentially expressed genes using the *clusterProfiler* package in R. All heat maps were drawn using the *Pheatmap* package in R.

### 2.7 Evaluation of immune cell fractions and immune subtypes

Myeloid‐derived suppressor cells (MDSCs),^25^ regulatory T cells (Tregs),^26^ natural killer (NK) cells^27^ and neutrophils^28^ in the glioma microenvironment are considered to be involved in immunosuppression. The ssGSEA method of the *GSVA* package was used to identify the immune cell composition mentioned above to evaluate the level of immune cell enrichment in the tumour microenvironment through the gene expression level in a single tumour sample.^29^ Vésteinn Thorsson and his colleagues categorized tumours into six immune subtypes, C1 (Wound Healing) has high angiogenic gene expression, high proliferation rate, and Th2 cells trending to the adaptive immune infiltrate; C2 (IFN‐γ Dominant) has the highest M1/M2 macrophage polarization level, a strong CD8 signal, and together with C6, the greatest T cell receptor diversity; C3 (Inflammatory) is determined by increased expression of Th17 and Th1 genes, low to moderate tumour cell proliferation, and, along with C5, lower aneuploidy levels and overall somatic copy number alterations compared with the other subtypes; C4 (Lymphocyte Depleted) displays a more prominent macrophage signature, with Th1 suppressed and a high M2 response; C5 (Immunologically Quiet) exhibits the lowest lymphocyte count, and highest macrophage responses, dominated by M2 macrophages; C6 (TGF‐β Dominant) displays the highest TGF‐β signature and a high lymphocytic infiltrate with an even distribution of Type I and Type II T cells. Subtype C4 is enriched in adrenocortical carcinoma (ACC), pheochromocytoma and paraganglioma (PCPG), hepatocellular carcinoma (LIHC) and gliomas, while low‐grade gliomas (LGG) consist mostly of subtype C5.^30^ The glioma samples were classified into different immune subtypes using the *ImmuneSubtypeClassifier* package in R.^30^

### 2.8 Independent prognostic role of the risk signature

To identify whether the ER stress‐related risk signature depended on other clinicopathological factors (including age, gender, tumour grade, IDH mutation status, 1p19q codeletion status and MGMT promoter methylation status) in predicting patients’ OS, univariate and multivariate Cox regression analyses were performed using the *survival* R package. The results of independent prognostic factor analysis were displayed in the form of forest plots using the *forestplot* R package.

### 2.9 Development and assessment of the nomogram

The nomogram shows the probability of clinical events through simple graphs of statistical prediction models to form a personalized prediction model. Age, gender, tumour grade, IDH mutation status, 1p19q codeletion status, MGMT promoter methylation status and ER stress‐related risk scores were combined to develop a nomogram using the R packages *survival* and *rms*. Calibration curves and decision curve analysis (DCA) were adopted to evaluate the accuracy of the nomogram in predicting one‐, three‐ and five‐year survival rates of glioma patients.^31^, ^32^ For the calibration curve, the higher was the degree of agreement between the predicted curve and the actual curve, the stronger was the predictive ability of the nomogram. For DCA, the decision curve of the nomogram was compared with that of other independent prognostic factors.

### 2.10 Statistical analyses

R software (version 3.6.3) and GraphPad Prism v7.00 (GraphPad Software Inc.) were used as statistical analysis tools in this study. Quantitative data are presented as the mean ± standard error of the mean (SEM) or standard deviation (SD). The Wilcoxon test was applied to compare the statistical differences between the two groups, and the Kruskal‐Wallis H test was employed to compare multiple groups. Statistical significance was defined as *P* < .05. Venn diagrams were drawn using jvenn.^33^ The other plots were constructed using R software or GraphPad Prism.

## 3 RESULTS

### 3.1 Sixteen ER stress‐related genes were identified to develop risk model

**FIGURE 1:**
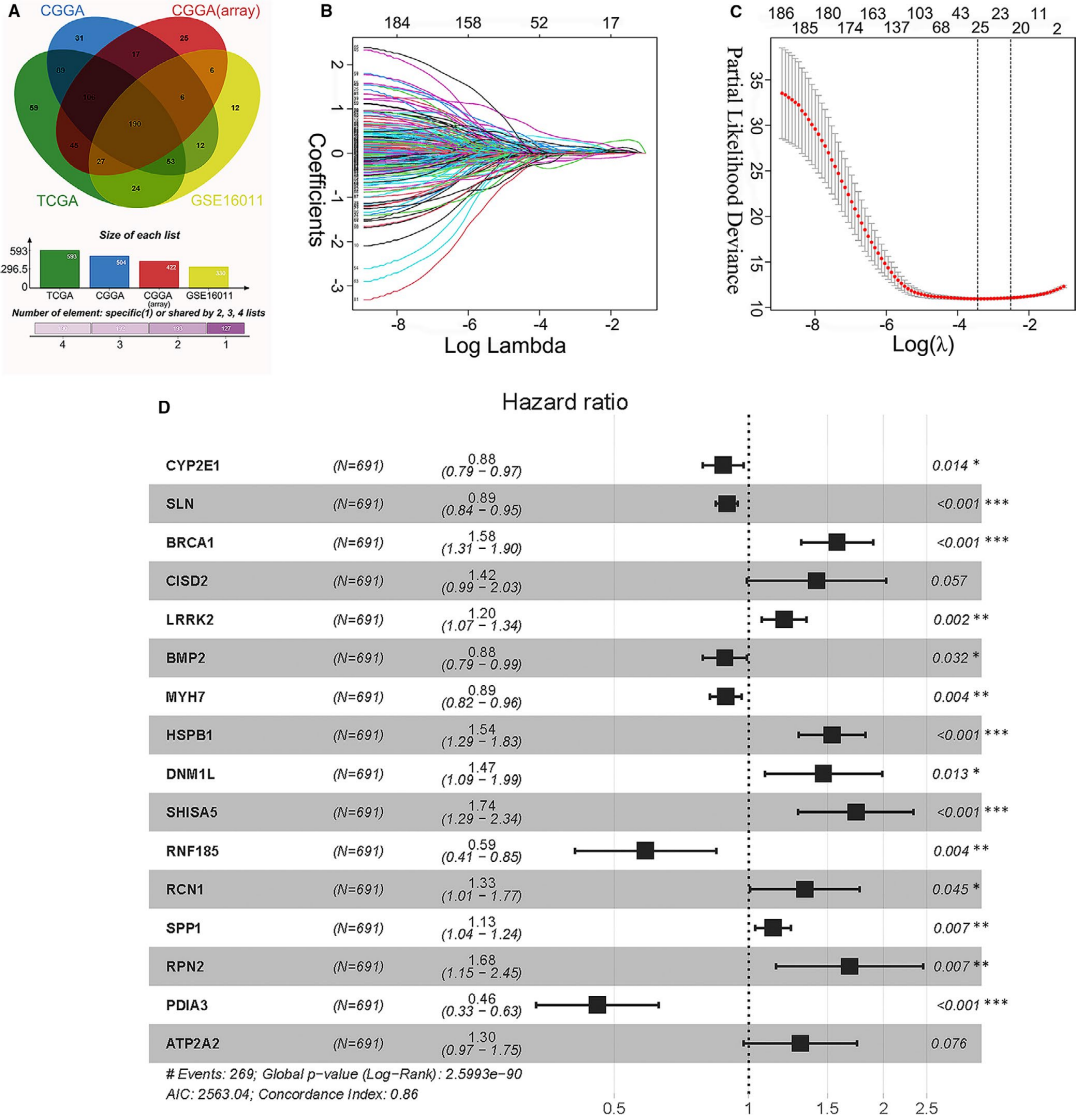
Identifying prognostic genes for developing a risk model. (A) Intersecting genes associated with glioma OS in the TCGA, CGGA, CGGA (mRNA‐array) and GSE160011 databases. (B) LASSO coefficient profiles of the 190 genes in the TCGA data set. (C) Selection of the optimal parameter (lambda) in the LASSO model. (D) Sixteen genes were chosen for establishing a prognosis signature. CGGA, Chinese Glioma Genome Atlas; LASSO, least absolute shrinkage and selection operator; OS, overall survival; TCGA, The Cancer Genome Atlas

A total of 787 ER stress‐related genes with a relevance score of ≥7 were extracted from the GeneCards database to generate prognostic gene signatures (Table S4). These genes were subjected to univariate Cox regression and KM analyses. In the TCGA, CGGA, CGGA (mRNA‐array) and GSE16011 data sets, 593, 504, 422 and 330 genes, respectively, were significantly related to OS of glioma patients (Figure 1A, Tables S5‐8). The overlapping genes (190 genes, Table S9) were included in the LASSO regression analysis to avoid overfitting problems in the risk signature (Figure 1B,C, Table S10). The AIC method of multivariate Cox regression analysis was applied to the genes returned from the LASSO regression analysis (28 genes) to construct the optimal model, which included sixteen genes (CYP2E1, SLN, BRCA1, CISD2, LRRK2, BMP2, MYH7, HSPB1, DNM1L, SHISA5, RNF185, RCN1, SPP1, RPN2, PDIA3 and ATP2A2) (Figure 1D). Among these genes, CYP2E1, SLN, BMP2, MYH7, RNF185 and PDIA3 were protective factors for glioma survival, with hazard ratios (HRs) <1, and BRCA1, CISD2, LRRK2, HSPB1, DNM1L, SHISA5, RCN1, SPP1, RPN2 and ATP2A2 were risk factors with HRs > 1.

Furthermore, we analysed the correlation of these 16 genes in the TCGA, CGGA and GSE16011 data sets and found that few genes were highly correlated with each other in the three data sets (Figure S1A‐C). We retrieved BRCA1, PDIA3, DNM1L, RCN1, HSPB1, RPN2, LRRK2 and SPP1 protein expression levels in gliomas using the Human Protein Atlas (HPA) (https://www.proteinatlas.org/). Expression levels of risk factors BRCA1, DNM1L, RCN1, HSPB1, RPN2, LRRK2 and SPP1 in high‐grade gliomas were greater than those in lower‐grade gliomas, while the protein expression levels of protective factor PDIA3 were exactly the opposite (Figure S2). We further examined the genetic alterations of these OS‐associated genes in glioma. A data set (http://www.cbioportal.org) comprising a merged cohort of low‐grade glioma (LGG) and GBM containing 794 patients/samples harbouring both mutations and CAN data was queried. Genetic alterations were found in 108 (13.60%) of the queried patients/samples (Figure S1D). CYP2E1 had the highest frequency of genetic alterations.

### 3.2 Establishing and evaluating the ER stress‐related risk signature

**FIGURE 2:**
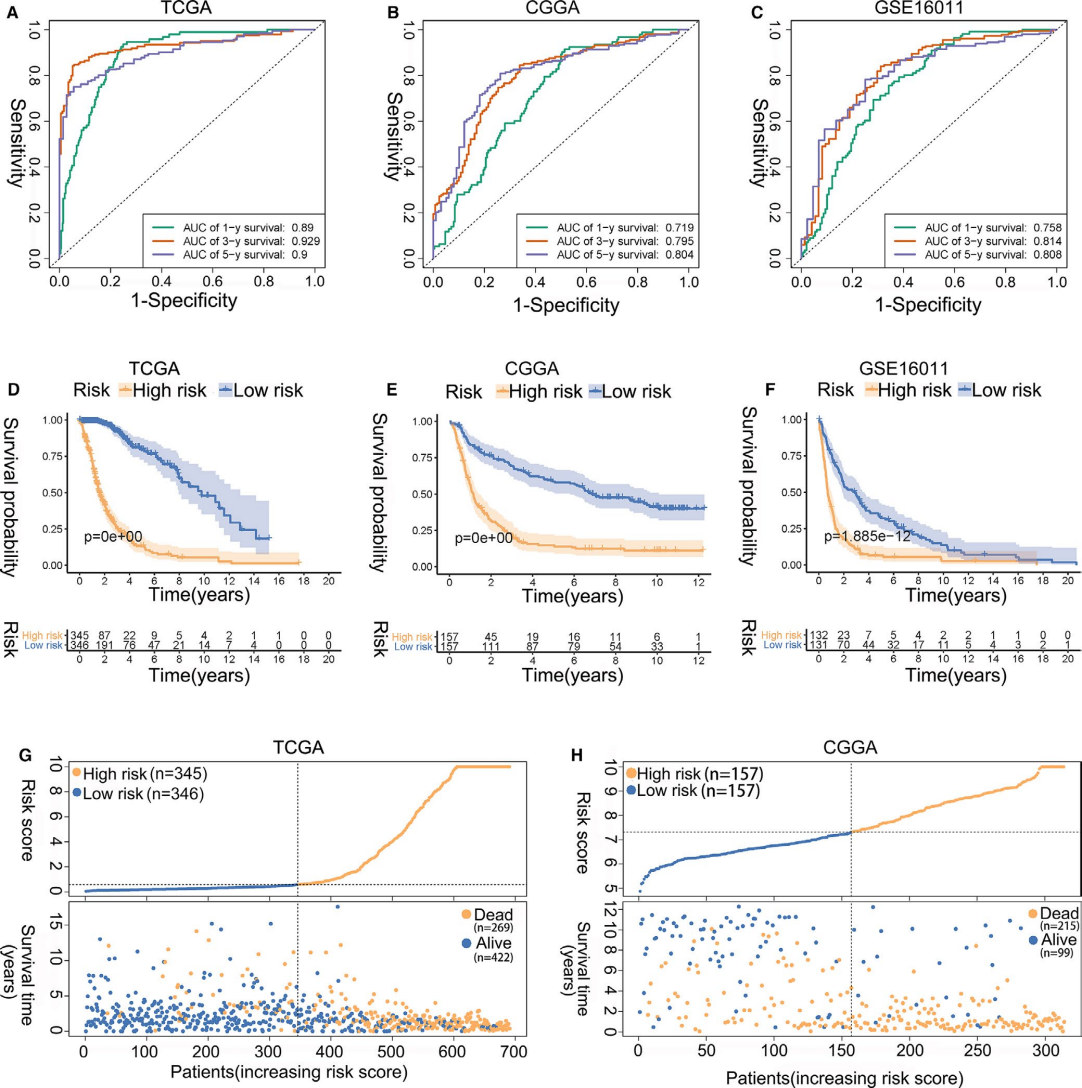
Assessment of the prognostic prediction ability of the ER stress‐related risk signature. (A‐C) ROC curves of the 16‐gene signature in the (A) TCGA, (B) CGGA and (C) GSE16011 cohorts. (D‐F) KM curve of the prognosis signature in the (D) TCGA, (E) CGGA and (F) GSE16011 cohorts (log‐rank test). (G, H) Risk score distribution in the (G) TCGA and (H) CGGA cohorts. CGGA, Chinese Glioma Genome Atlas; ER, Endoplasmic reticulum; LASSO, least absolute shrinkage and selection operator; ROC, receiver operating characteristic; TCGA, The Cancer Genome Atlas

The Cancer Genome Atlas glioma data were used to construct the risk signature. The risk score was calculated as follows: risk score = (−0.1318 × CYP2E1 expression) + (−0.1122 × SLN expression) + (0.4543 × BRCA1 expression) + (0.3481 × CISD2 expression) + (0.1811 × LRRK2 expression) + (−0.1236 × BMP2 expression) + (−0.1183 × MYH7 expression) + (0.4296 × HSPB1 expression) + (0.3839 × DNM1L expression) + (0.5514 × SHISA5 expression) + (−0.5315 × RNF185 expression) + (0.2889 × RCN1 expression) + (0.1260 × SPP1 expression) + (0.5187 × RPN2 expression) + (−0.7779 × PDIA3 expression) + (0.2649 × ATP2A2 expression). Time‐dependent ROC curves were used to assess the efficiencies of the prognostic prediction of the ER stress‐related risk signature. As presented in Figure 2A, the AUCs for 1‐, 3‐ and 5‐year OS in the TCGA data set were 0.890, 0.929 and 0.900, respectively. The AUCs for predicting 1‐, 3‐ and 5‐year OS in the CGGA data set were 0.719, 0.795 and 0.804, respectively, and those in the GSE16011 data set were 0.758, 0.814 and 0.808, respectively (Figure 2B,C).

Samples in the TCGA, CGGA and GSE16011 cohorts were then divided into low‐ and high‐risk groups based on the median risk score in each cohort. KM analysis showed that patients in the low‐risk group had a more favourable outcome than patients in the high‐risk group (Figure 2D‐F). The three‐year OS rates were 26.56% (95% confidence interval (CI): 21.20‐33.28) vs 92.43% (95% CI: 88.72‐96.32) for the high‐ and low‐risk groups in the TCGA data set, 22.38% (95% CI: 16.56‐30.25) vs 69.4% (95% CI: 62.40‐77.21) for the high‐ and low‐risk groups in the CGGA cohort, and 10.81% (95% CI: 6.60‐17.72) vs 47.88% (95% CI: 39.97‐57.37) for the high‐ and low‐ risk groups in the GSE16011 data set, respectively. Figure 2G‐H and Figure S3A show the OS‐related prediction model distribution of patients in the TCGA, CGGA and GSE16011 data sets. These results indicate the accuracy of the ER stress‐related risk signature in predicting the outcomes of glioma patients. The expression patterns of the 16 genes in the TCGA, CGGA and GSE16011 data sets are shown in Figure S3B‐D.

### 3.3 The ER stress‐related signature is associated with clinicopathological features

**FIGURE 3:**
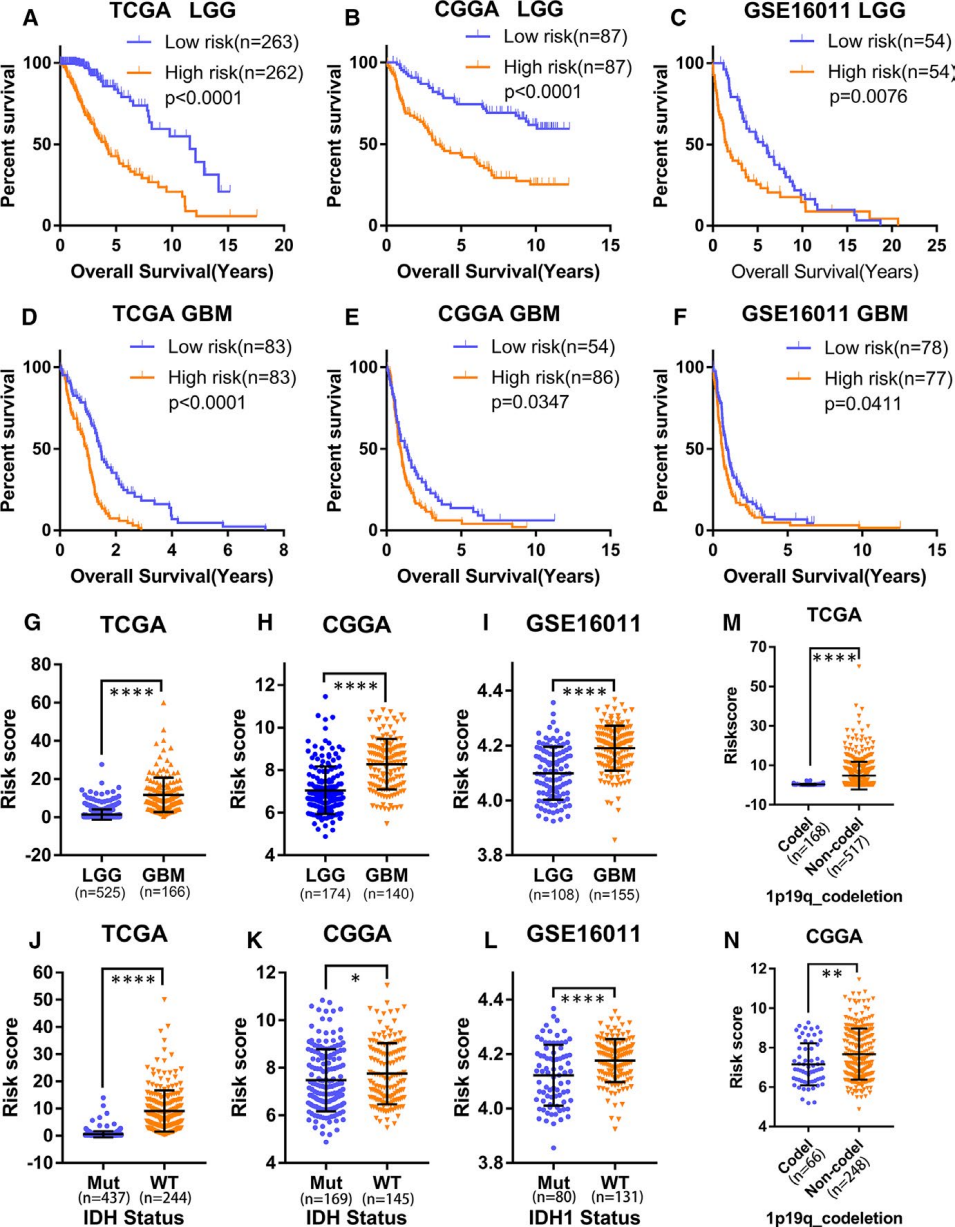
The ER stress risk score can distinguish different clinicopathological features of gliomas. (A‐C) The prognosis values of the ER stress risk model in LGG in the (A) TCGA, (B) CGGA and (C) GSE16011 data sets (log‐rank test). (D‐F) The prognosis values of the ER stress risk signature in GBM from the (D) TCGA, (E) CGGA and (F) GSE16011 data sets (log‐rank test). (G‐I) Distribution of the risk scores in LGG and GBM in the (G) TCGA, (H) CGGA and (I) GSE16011 data sets (Wilcoxon test). (J‐N) The risk score was grouped by IDH mutation status (J‐L) and 1p19q codeletion status (M and N) (Wilcoxon test). CGGA, Chinese Glioma Genome Atlas; ER, Endoplasmic reticulum; IDH, isocitrate dehydrogenase; LGG, low‐grade gliomas; ROC, receiver operating characteristic; TCGA, The Cancer Genome Atlas

We investigated whether the ER stress‐related risk signature was correlated with the clinicopathological features of glioma patients. First, we examined the prognostic effect of the signature in LGG and GBM patients using KM analysis. As displayed in Figure 3A‐F, the high‐risk group showed reduced OS in terms of both LGG and GBM in the TCGA, CGGA and GSE16011 data sets. Additionally, the risk scores were notably different among stratified patients, with high‐risk scores in high‐grade glioma, wild‐type IDH, 1p19q non‐codeletion, mesenchymal subtype and MGMT promoter unmethylated patients (Figure 3G‐N, Figure S4A‐D). These results suggest that the ER stress‐related risk signature can accurately distinguish different clinicopathologic features of glioma patients.

### 3.4 Functional annotation of the risk signature

**FIGURE 4:**
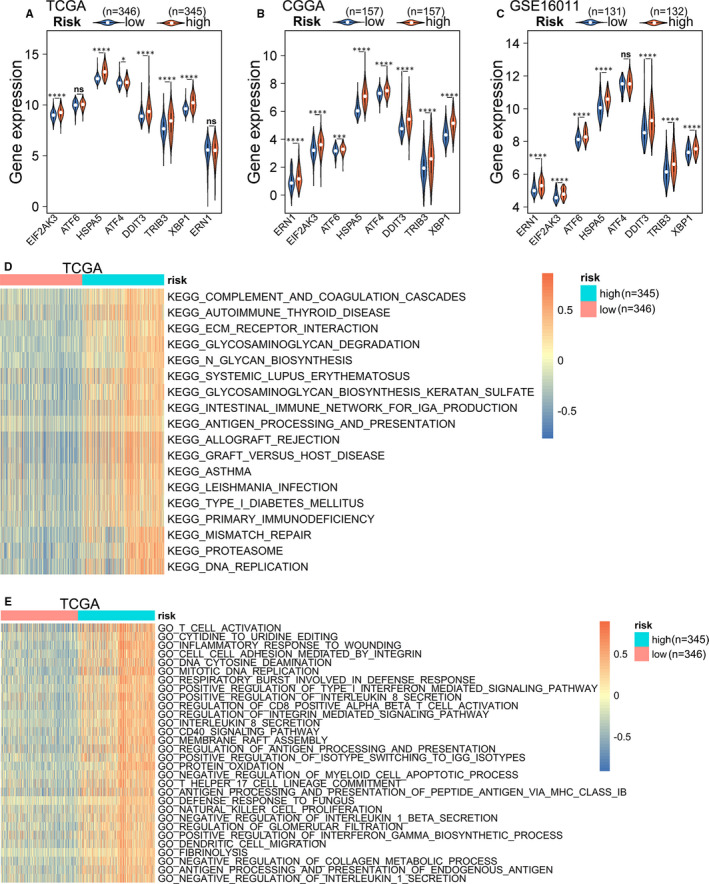
Functional enrichment analysis of the risk signature. (A‐C) Differential expression of ER stress markers in the (A) TCGA, (B) CGGA, and (C) GSE16011 between different groups (Wilcoxon test). (D) The top 20 KEGG pathways and (E) the top 30 biological processes enriched in the high‐risk group in the TCGA cohort. CGGA, Chinese Glioma Genome Atlas; ER, Endoplasmic reticulum; KEGG, Kyoto Encyclopedia of Genes and Genomes; TCGA, The Cancer Genome Atlas

Many studies on ER stress have used the intracellular expression levels of related proteins, such as ATF6, HSPA5, XBP1 and ATF4, as indicators to examine the intensity of ER stress in cells or tissues.^34^, ^35^, ^36^ To explore ER stress status in different glioma groups, we measured the expression levels of these markers in the TCGA, CGGA and GSE16011 data sets. Most of them had higher expression levels in the high‐risk group, demonstrating that the ER stress in this group was markedly more intense than that in the low‐risk group (Figure 4A‐C). To further verify the relationship between the risk score and the ER stress intensity of gliomas, we used qRT‐PCR to measure the expression levels of the 16 risk genes in the 12 glioma samples. The samples were scored based on the expression levels of these genes and grouped according to the score. We then used Western blotting to measure the expression levels of ATF6, EIF2α, p‐EIF2α and p‐IRE1α in the samples from the high‐ and low‐score groups. The Western blotting results were consistent with those of the above databases analysis (Table S11, Figure S5). These results further confirmed the correlation between the signature and ER stress activation. Next, we used the *GSVA* package to explore the KEGG pathways and GO biological processes associated with risk signature in the TCGA and CGGA data sets. At *P* < .05, the top 20 KEGG pathways and the top 30 GO biological processes were identified based on the logFC value in the TCGA and CGGA data sets. Most of the KEGG pathways and GO biological processes enriched in the high‐risk group were associated with the immune and inflammatory responses as well as cell biosynthesis and degradation (Figure 4D,E, Figure S6A,B). To confirm these results, differentially expressed genes between the two groups were selected and underwent GO and KEGG analyses using the R package, *clusterProfiler*, in the TCGA, CGGA and GSE16011 data sets (Figure S7A‐F). The results were similar to the results of the GSVA analysis.

The typical characteristic of ER stress is cellular protein synthesis in excess of the ER folding ability. This may be why biosynthesis‐ and degradation‐related pathways were enriched in the high‐risk groups. In addition, studies have confirmed that ER stress inhibits anti‐tumour immunity and promotes tumour cell escape from immunosurveillance.^13^, ^37^, ^38^ Here, the enrichment in immune and inflammatory response‐related pathways and processes in the high‐risk group further demonstrates that the immune environment of the high‐risk group is complex.

### 3.5 High ER stress‐related risk score demonstrates an immunosuppressive feature

**FIGURE 5:**
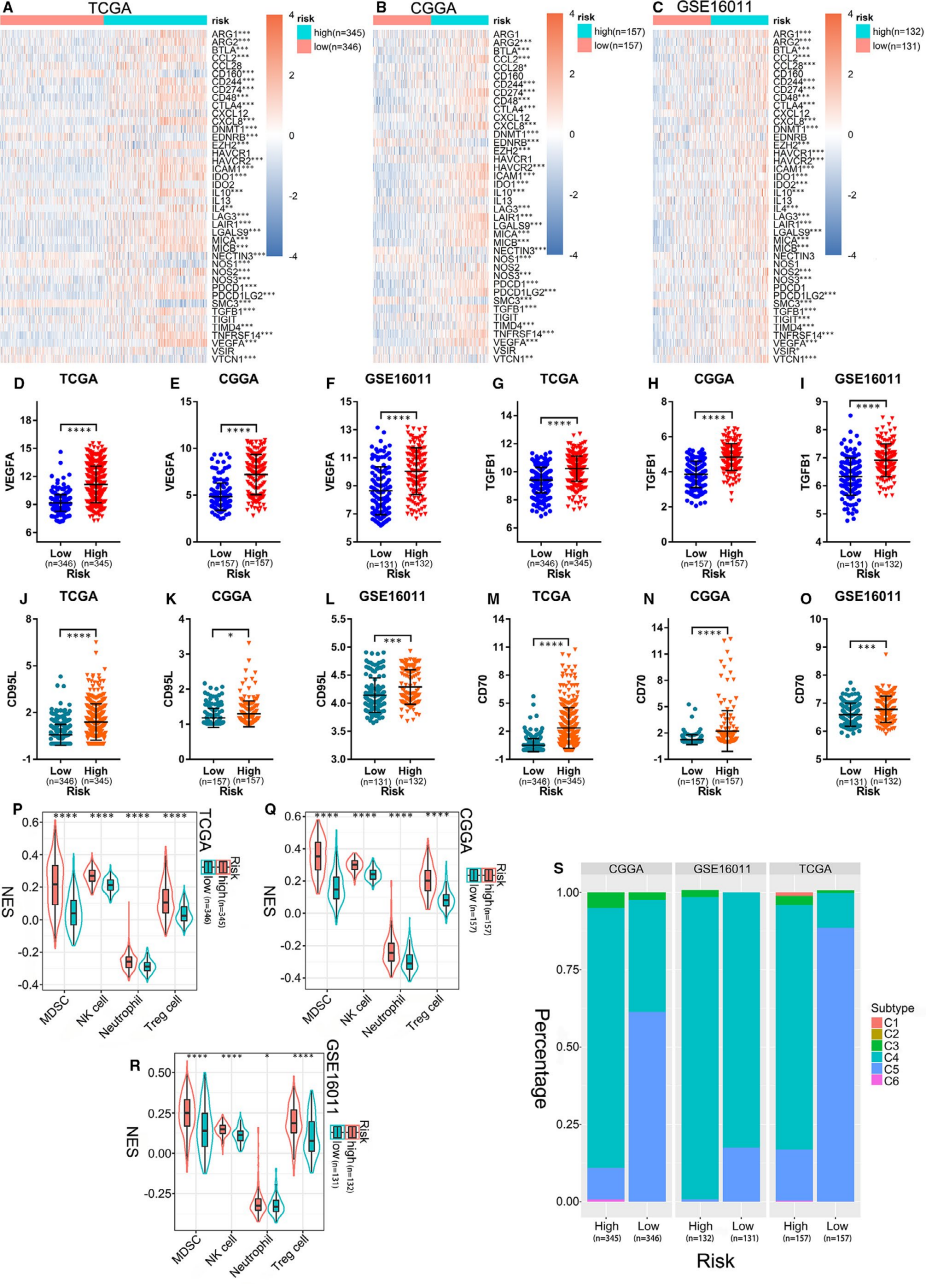
Immune features between different groups of glioma patients. (A‐C) Expression of the cancer‐immunity cycle negative regulators in low‐ and high‐risk groups in the (A) TCGA, (B) CGGA and (C) GSE16011 data sets (Wilcoxon test). (D‐F) VEGFA, (G‐I) TGFB1, (J‐L) CD95L and (M‐O) CD70 expression in the low‐ and high‐risk groups (Wilcoxon test). (P‐R) Immunosuppressive cell scores in different groups (Wilcoxon test). (S) Distribution of immune subtypes. CGGA, Chinese Glioma Genome Atlas; TCGA, The Cancer Genome Atlas

The process of tumour eradication by the immune system involves the cancer‐immunity cycle.^39^ We explored the expression characteristics of genes that inhibited this cycle in the TCGA, CGGA and GSE16011 data sets. These genes were acquired from the Tracking Tumor Immunophenotype website (http://biocc.hrbmu.edu.cn/TIP/index.jsp).^40^ In the three cohorts, most genes were highly expressed in the high‐risk group (Figure 5A‐C). TGFB1, VEGFA, ARG1, FGL2 and IL10 are secreted immunosuppressive factors in glioma,^41^, ^42^, ^43^, ^44^, ^45^, ^46^ while CD95L and CD70 are glioma cell‐surface immunosuppressive factors.^47^, ^48^ As shown in Figure 5D‐O and Figure S8A‐I, they were all obviously overexpressed in the high‐risk groups. Furthermore, some immune cells, such as MDSCs, Tregs, NK cells, and neutrophils, infiltrate into the tumour microenvironment and exhibit immunosuppressive effects to promote tumour occurrence, progression, and therapy resistance.^25^, ^26^, ^27^, ^28^ These immunosuppressive cells were also found to be enriched in the ER stress‐related high‐risk groups (Figure 5P‐R). To further explore the characteristics of the immune microenvironment in glioma of the high‐ and low‐risk groups, the *ImmuneSubtypeClassifier* package was used to divide the samples into different immune subtypes in the three cohorts. We found that in both the high‐ and the low‐risk groups, the main subtypes were C4 and C5, but the high‐risk group had considerably more C4 subtypes than the low‐risk group (Figure 5S). The prognosis of the C4 immune subtype in tumours is worse than that of the C5 immune subtype,^30^ which was consistent with the prognosis of high‐ and low‐risk glioma patients. This further confirmed the accuracy of the ER stress‐related risk signature in predicting immune subtypes and prognosis in gliomas.

**FIGURE 6:**
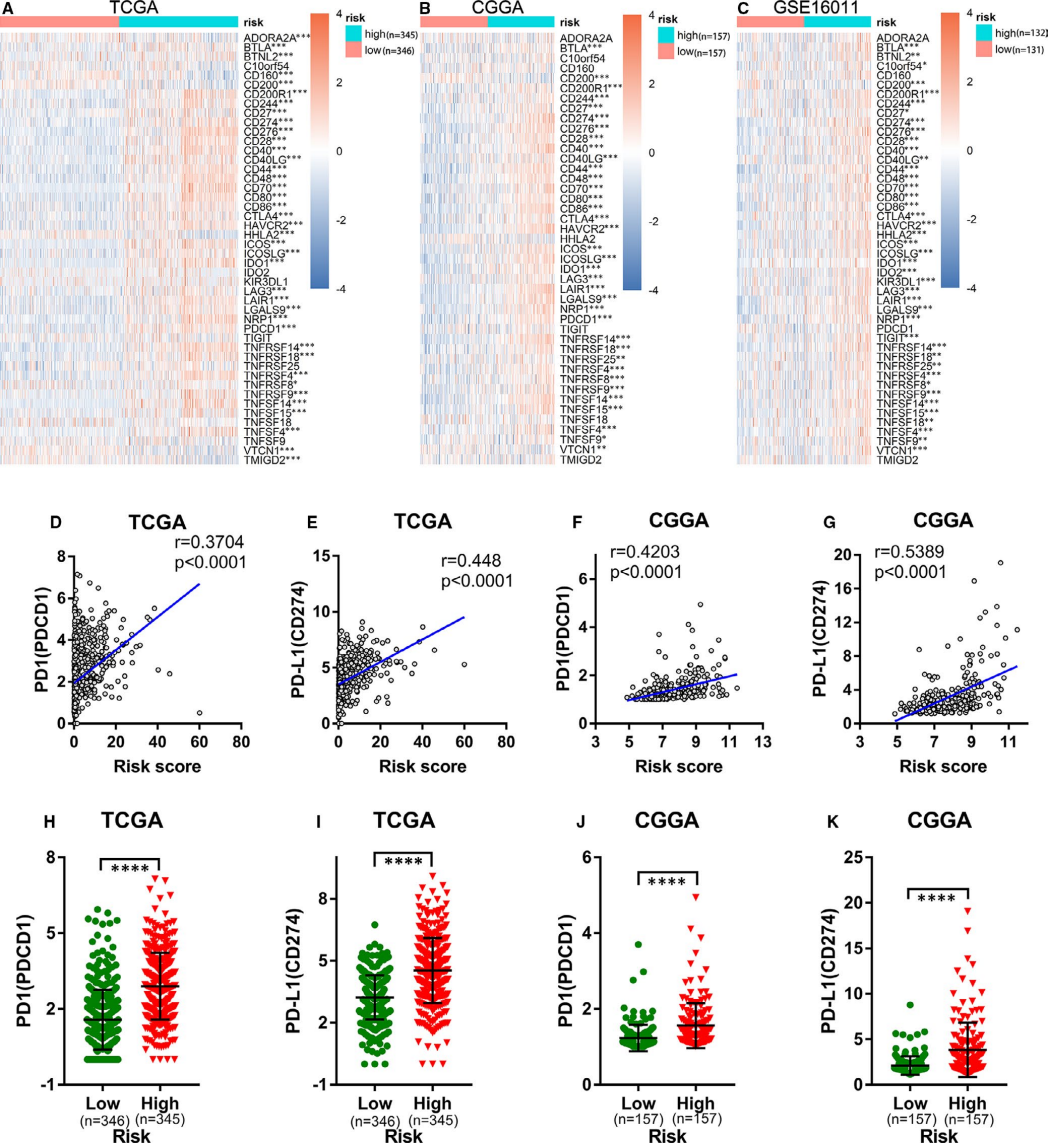
Relationship between the ER stress risk signature and immune checkpoints. (A‐C) Heat map of immune checkpoints in the low‐ and high‐risk groups in the (A) TCGA, (B) CGGA and (C) GSE16011 databases (Wilcoxon test). (D, E) Correlation between the ER stress risk score and the expression of (D) PD1 and (E) PD‐L1 in the TCGA cohort (Pearson correlation analysis). (F, G) Correlation between the ER stress risk score and the expression of (F) PD1 and (G) PD‐L1 in the CGGA cohort (Pearson correlation analysis). (H and J) PD1 and (I and K) PD‐L1 expression in different groups in the TCGA and CGGA data sets (Wilcoxon test). CGGA, Chinese Glioma Genome Atlas; ER, Endoplasmic reticulum; TCGA, The Cancer Genome Atlas

In addition to the cancer‐immunity cycle inhibitors mentioned above, immune checkpoints can also suppress the immune system's ability to clear tumours.^49^ In recent years, immune checkpoints have become potential therapeutic targets for many malignant tumours and play an important role in tumour immunotherapy.^50^ Comparison of the expression of immune checkpoints in the high‐ and low‐risk groups showed that most immune checkpoints were up‐regulated in the high‐risk groups in the TCGA, CGGA and GSE16011 cohorts (Figure 6A‐C). Considering the critical roles played by PD1 and PD‐L1 in tumour immunosuppression and immunotherapy, we separately investigated the relationship between their expression levels and the ER stress‐related risk score. We found that the expression levels of PD1 and PD‐L1 were obviously positively correlated with the risk score (Figure 6D‐G), and their expression levels in the high‐risk group were markedly higher than those in the low‐risk group (Figure 6H‐K). These data suggest that the ER stress‐related risk signature can accurately predict the immune characteristics of glioma.

### 3.6 Construction and validation of the nomogram

**FIGURE 7:**
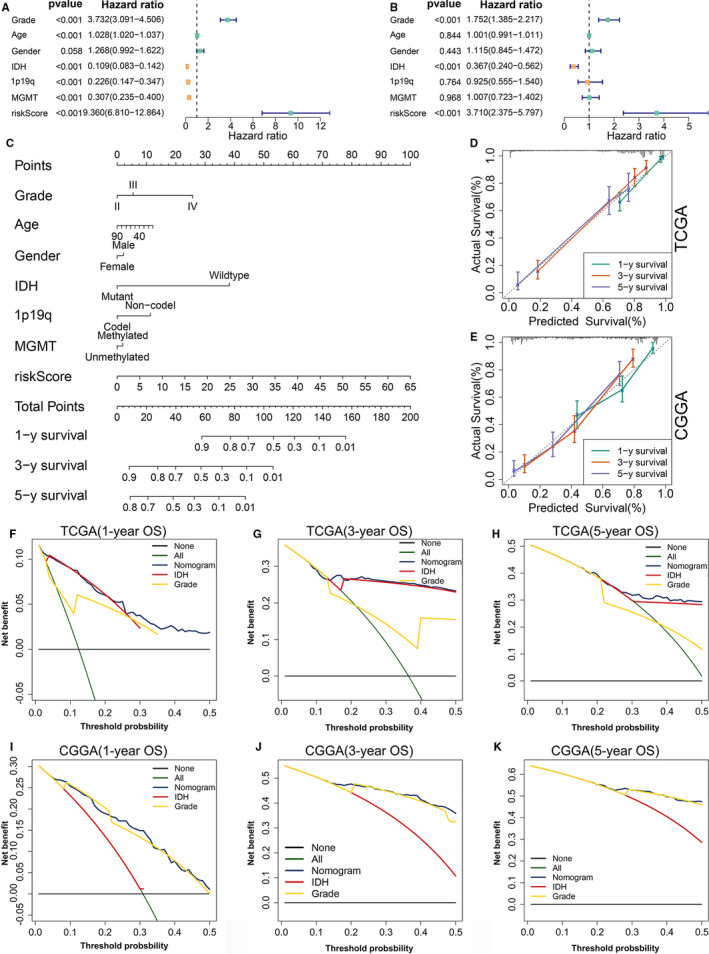
Establishment and assessment of the nomogram. (A, B) Forest plot of the (A) univariate and (B) multivariate Cox regression analyses in the TCGA cohort. (C) The nomogram plot was constructed based on WHO grade, age, gender, IDH mutation status, 1p19q codeletion status, MGMT promoter methylation status and ER stress risk score. (D, E) Calibration plot of the nomogram based on (D) TCGA and (E) CGGA data. (F‐H) DCA of the nomogram for 1‐, 3‐ and 5‐year OS in the TCGA cohort. (I‐K) DCA of the nomogram for 1‐, 2‐ and 5‐year OS in the CGGA cohort. CGGA, Chinese Glioma Genome Atlas; DCA, decision curve analysis; ER, Endoplasmic reticulum; IDH, isocitrate dehydrogenase; OS, overall survival; TCGA, The Cancer Genome Atlas

In addition to the ER stress risk score, there are numerous known prognostic factors for glioma, such as age, gender, WHO grade, IDH mutation status, 1p19q codeletion status and MGMT promoter methylation status. Therefore, it was necessary to examine whether the ER stress risk signature could independently predict prognosis. In the TCGA training cohort, univariate Cox analysis revealed that ER stress‐related risk score was negatively correlated with the OS of glioma patients. Moreover, age, grade, IDH mutation status, 1p19q codeletion status and MGMT promoter status were also significantly related to OS (Figure 7A). Subsequent multivariate Cox regression analysis indicated that the ER stress‐related risk signature, grade and IDH mutation status were significantly correlated with OS (Figure 7B). These results were further confirmed in the CGGA and GSE16011 data sets (Figure S9A‐D). These findings indicate that the ER stress‐related risk signature constructed using the TCGA data set was an independent prognostic factor for glioma patients.

We established a nomogram to predict 1‐, 3‐ and 5‐year OS in the TCGA data set, by integrating ER stress risk signature, age, gender, WHO grade, IDH mutation status, 1p19q codeletion status and MGMT promoter methylation status. In the nomogram, each signature was assigned points according to its risk contribution to OS (Figure 7C). The calibration curves illustrated a remarkable consensus between the predicted and actual survival time in terms of the 1‐, 3‐ and 5‐year OS rates in the TCGA and CGGA cohorts (Figure 7D,E). The DCAs constructed using the TCGA (Figure 7F‐H) and CGGA (Figure 7I‐K) cohorts for this nomogram demonstrated that the nomogram performed well in predicting the 1‐, 3‐ and 5‐year OS rates in glioma patients.

## 4 DISCUSSION

Substantial data suggest that a specific intensity of ER stress promotes multiple mechanisms of cancer progression, including cancer cell survival and metastasis, therapeutic resistance, and angiogenesis.^11^, ^12^ ER stress can help cancer cells evade immunity and facilitate metastases.^38^ Moreover, ER stress in tumour cells can also affect tumour immunity. Cancer cells under ER stress release unknown factors to induce ER stress in macrophages in the microenvironment, inducing them to release pro‐inflammatory factors. Simultaneously, these unknown factors also promote bone marrow‐derived dendritic cells to release immunosuppressive factors, such as Arginase, to inhibit the antigen presentation ability of CD8^+^ T cells.^13^, ^37^ In breast cancer, ER‐stressed tumour cells can up‐regulate miR‐27a‐3p content in exosomes and promote PD‐L1 expression in macrophages.^51^ Although ER stress in tumour cells has the afore‐mentioned effects on immune cells in the tumour microenvironment, the specific mechanism has not yet been fully elucidated. One possible explanation is that ER‐stressed tumour cells can induce ER stress in immune cells in the tumour microenvironment. Due to ER stress signalling, such as IRE1α, and PERK, the function of ER‐stressed immune cells is inhibited.^13^, ^52^, ^53^ Collectively, these findings indicate that a complete understanding of the specific mechanisms of ER stress in the regulation of anti‐tumour immunity may greatly improve the efficiency of tumour immunotherapy.

In gliomas, ER stress has an important influence on tumorigenesis, progression and therapy response.^54^ However, the role of ER stress in the anti‐tumour immunity of glioma remains unclear. Based on the content presented above, we speculated that ER stress might affect immune characteristics within the glioma microenvironment.

In this study, we retrieved and downloaded 787 ER stress‐related genes from the GeneCards website. Among them, 190 survival‐related genes were shared among the TCGA, CGGA, CGGA (mRNA‐array) and GSE16011 data sets. Further LASSO regression analysis and AIC of multivariate Cox regression analyses were performed to identify16 OS‐related genes (CYP2E1, SLN, BRCA1, CISD2, LRRK2, BMP2, MYH7, HSPB1, DNM1L, SHISA5, RNF185, RCN1, SPP1, RPN2, PDIA3 and ATP2A2) and construct an OS‐related prediction model. CYP2E1, SLN, BMP2, MYH7, RNF185 and PDIA3 expression levels were positively correlated with favourable outcomes, whereas BRCA1, CISD2, LRRK2, HSPB1, DNM1L, SHISA5, RCN1, SPP1, RPN2 and ATP2A2 expression levels were negatively correlated with favourable outcomes.

Of the 16 OS‐related genes, CYP2E1,^55^ PDIA3,^56^ RNF185,^57^ CISD2^58^ and DNM1L^59^ are up‐regulated and promote cancer progression. Additionally, CYP2E1 regulates the level of ER stress and reactive oxygen species,^60^ PDIA3 is closely related to the anti‐tumour immunity of glioma,^61^ and DNM1L plays a regulatory role in the immune response of NKT cells to tumours.^62^ The role of SHISA5 and SLN in cancers has not been previously studied. BRCA1 mutations are closely related to the tumorigenesis and progression of female breast and ovarian tumours.^63^ In hepatocellular carcinoma, BRCA1 expression level is positively correlated with the infiltration levels of immune cells including B cells, CD8^+^ T cells, macrophages and dendritic cells.^64^ HSPB1 can negatively regulate the ferroptotic death of tumour cells.^65^ Silencing HSPB1 in breast cancer cells can enhance the cytotoxicity of CD8^+^ T cells and their transformation into memory cells.^66^ BMP2 is down‐regulated in colorectal cancer tissues while also being able to suppress colorectal cancer progression.^67^ In osteogenesis, BMP2 is a potential chemokine for macrophages and significantly reduces M1 phenotypic markers, including IL‐1β and IL‐6, in macrophages.^68^ Thus, it is not unexpected that most of the 16 genes identified directly or indirectly affect the function of immune cells. This also suggests that ER stress may regulate the anti‐glioma immune response.

Risk score is a commonly employed method for the development of a meaningful signature. The model we built using ER stress‐related risk scores not only accurately predicted the prognosis of glioma patients, but also distinguished different glioma molecular subtypes. ROC analysis demonstrated that the 16‐gene signature performed well in predicting short‐term (1‐year and 3‐year) and long‐term (5‐year) survival for glioma patients in the TCGA, CGGA and GSE16011 data sets. KM analysis confirmed that the model accurately predicted the survival of glioma patients.

Considering the powerful role of this risk signature in gliomas, we further evaluated the mechanisms of these effects. Functional analysis suggested that the biological processes of immune and inflammatory responses, as well as biosynthesis and degradation, were enriched in the high‐risk group, suggesting an interaction between ER stress and the glioma immune response. Compared with the low‐risk group, the high expression of cancer‐immunity cycle inhibitors and immune checkpoints along with the enrichment of tumour‐immunosuppressive cells in the high‐risk group indicated that the model successfully differentiated the immune types of glioma. This indicates that ER stress can regulate the immune microenvironment of glioma to affect the prognosis of glioma patients. It also confirms that our assumption regarding the relationship between ER stress and the anti‐glioma immune response is correct.

To fully utilize the potential of the risk model, we developed a nomogram combining the ER stress signature, age, gender, WHO grade, IDH mutation status, 1p19q codeletion status and MGMT promoter methylation status. Calibration plots and DCA based on the TCGA and CGGA databases demonstrated the excellent predictive performance of the nomogram. Thus, our 16‐gene ER stress‐related risk signature can predict the OS of glioma patients and facilitate the selection of optimal treatment approaches.

However, our study also had some limitations. The expression and prognostic predictive effect of the 16 genes at the protein level require additional assessment. In addition, further research is needed to confirm the specific functional mechanisms of the ER stress‐related risk signature in glioma. Although it showed outstanding performance in distinguishing glioma survival differences and immune characteristics, the accuracy of the risk model in discriminating between normal brain tissue and glioma tissue remains to be investigated. In summary, our research provides important resources for elucidation of the specific role of ER stress in glioma.

## CONFLICT OF INTEREST

The authors declare that the research was conducted in the absence of any commercial or financial relationships that could be construed as a potential conflict of interest.

## AUTHOR CONTRIBUTIONS

**Qing Zhang:** Conceptualization (equal); Data curation (equal); Investigation (equal); Methodology (equal); Project administration (equal); Software (equal); Writing‐original draft (equal). **Gefei Guan:** Methodology (equal); Visualization (equal). **Peng Cheng:** Methodology (equal); Supervision (equal); Validation (equal). **Wen Cheng:** Methodology (equal); Supervision (equal); Validation (equal). **Lian‐He Yang:** Methodology (equal); Validation (equal). **Anhua Wu:** Conceptualization (equal); Data curation (equal); Supervision (equal); Validation (equal); Writing‐original draft (equal).

## Supporting information

Figure S1‐S9

Table S1

Table S2

Table S3

Table S4‐S11

## Data Availability

The data sets used in this study can be downloaded from http://www.cgga.org.cn/ (CGGA), https://portal.gdc.cancer.gov/ (TCGA), and https://www.ncbi.nlm.nih.gov/geo/query/acc.cgi?acc=GSE16011 (GSE16011).
