# Supplementary material for: Characterization of an endoplasmic reticulum stress‐related signature to evaluate immune features and predict prognosis in glioma
Source: J Cell Mol Med. 2021 Feb 21;25(8):3870–84. doi: 10.1111/jcmm.16321 (PMC8051731; doi:10.1111/jcmm.16321)
Supplement: Supplementary file 1 — Figure S1‐S9 [file JCMM-25-3870-s005.docx]

**Supplementary Figures**


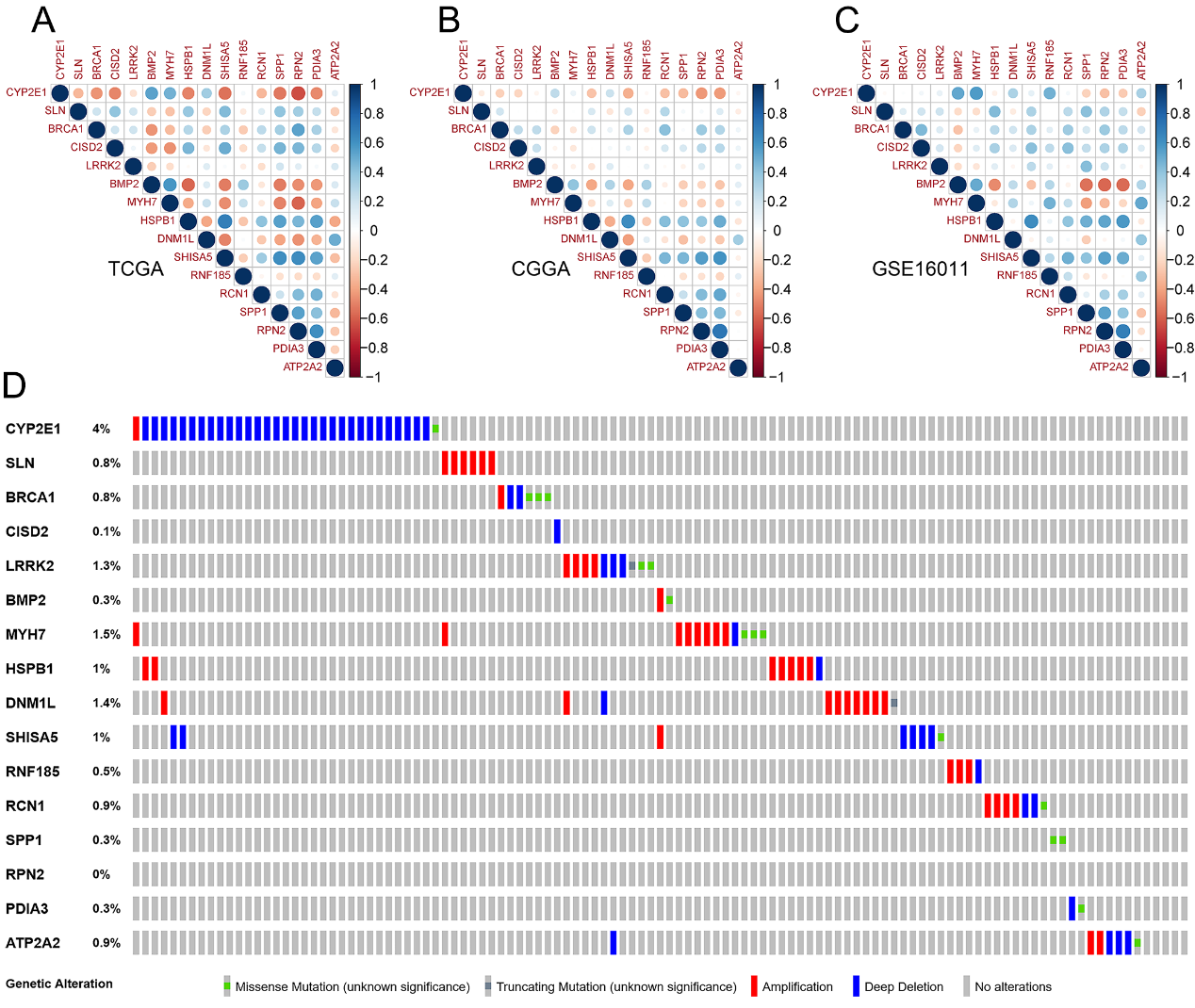


**Supplementary Figure 1.** Correlation between the 16 genes and their genetic alteration status. (A−C) Spearman correlation analysis of 16 ER stress-related genes in the TCGA, CGGA, and GSE16011 datasets. (D) Genetic alteration of the 16 genes in the TCGA LGG and GBM cohort.


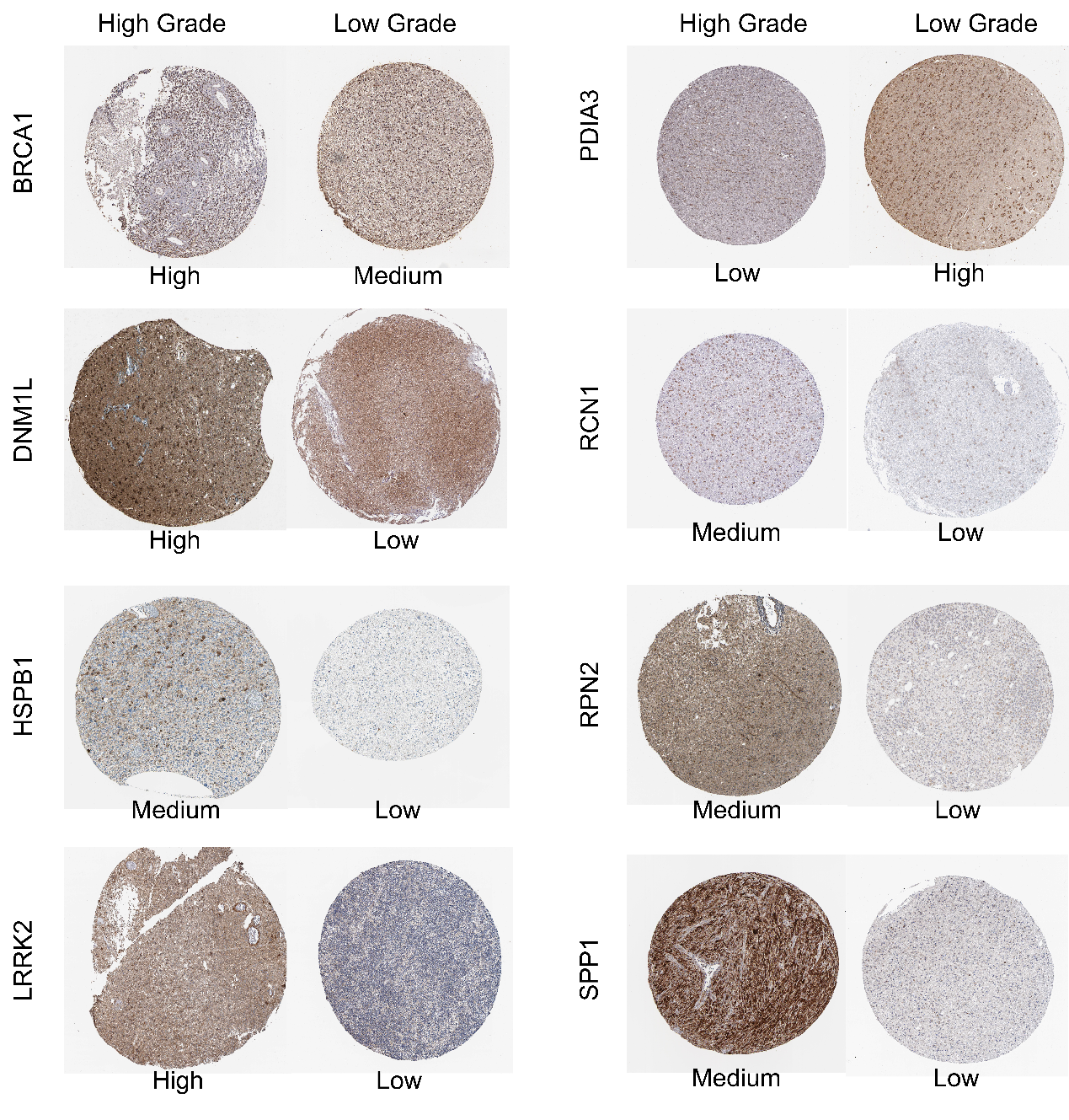


**Supplementary Figure 2:** The expression profiles of the proteins encoded by BRCA1, PDIA3, DNM1L, RCN1, HSPB1, RPN2, LRRK2, and SPP1 in low- and high-grade glioma tissues using clinical specimens from the Human Protein Profiles.


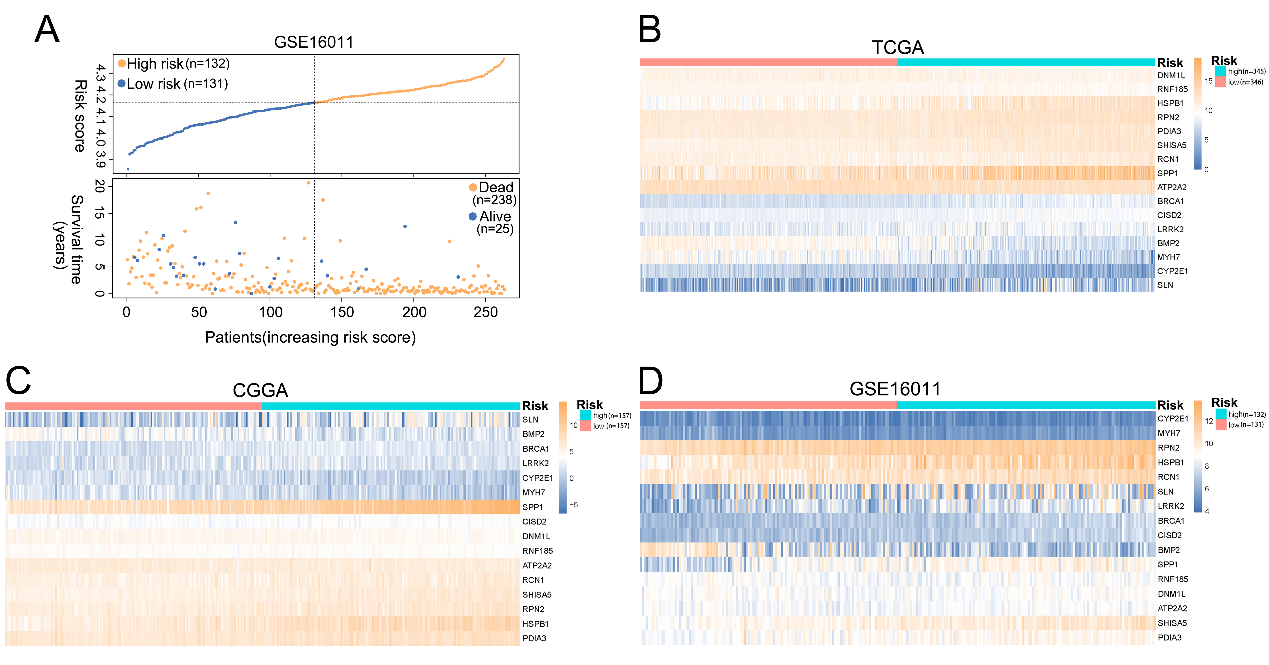
**Supplementary Figure 3.** The expression pattern of the 16 genes. (A) Risk score of the 16-gene signature in the GSE16011 cohort. (B−D) Heatmap of mRNA expression of the 16-gene signature in (B) TCGA, (C) CGGA, and (D) GSE16011 datasets.


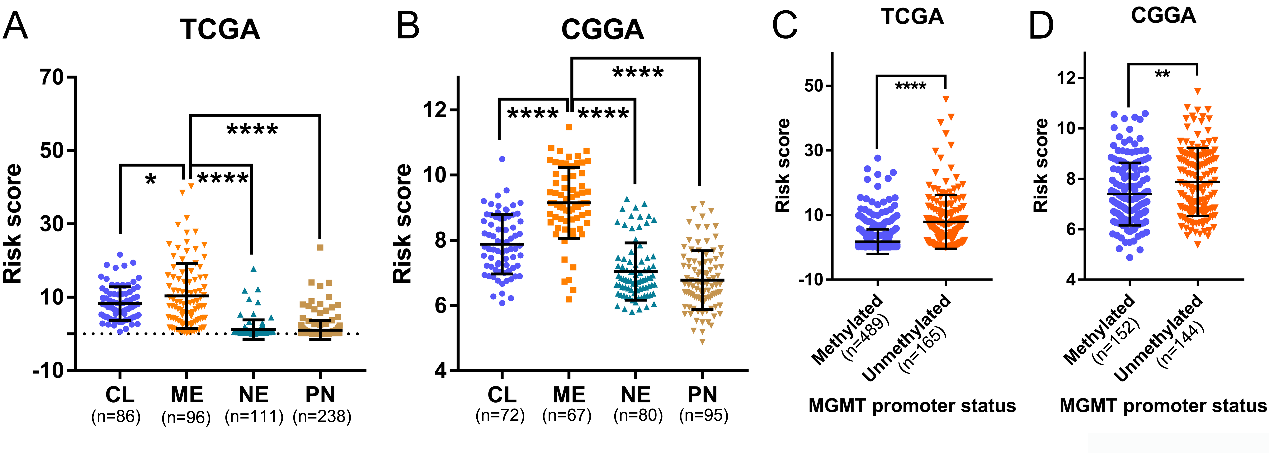
**Supplementary Figure 4.** (A−B) The distribution of risk scores in glioma subtypes in the (A) TCGA and (B) CGGA cohorts (Wilcoxon test). (C−D) The distribution of risk scores in MGMT promoter methylated and unmethylated samples in the (C) TCGA and (D) CGGA cohorts (Wilcoxon test).


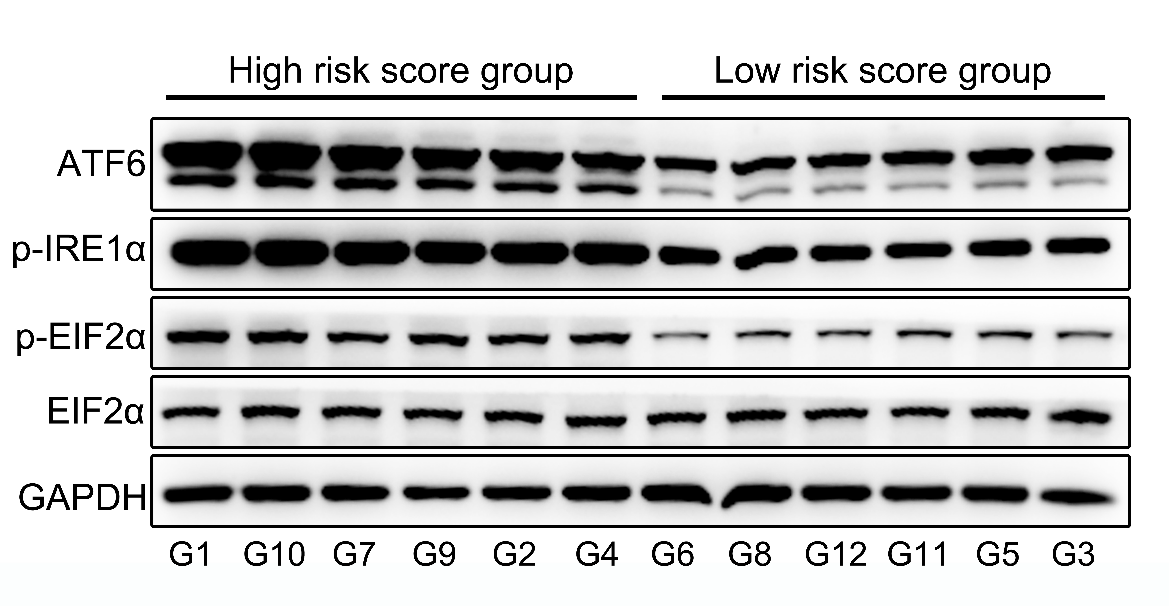


**Supplementary Figure 5.** The expression of ER stress markers in high- and low-risk glioma samples.


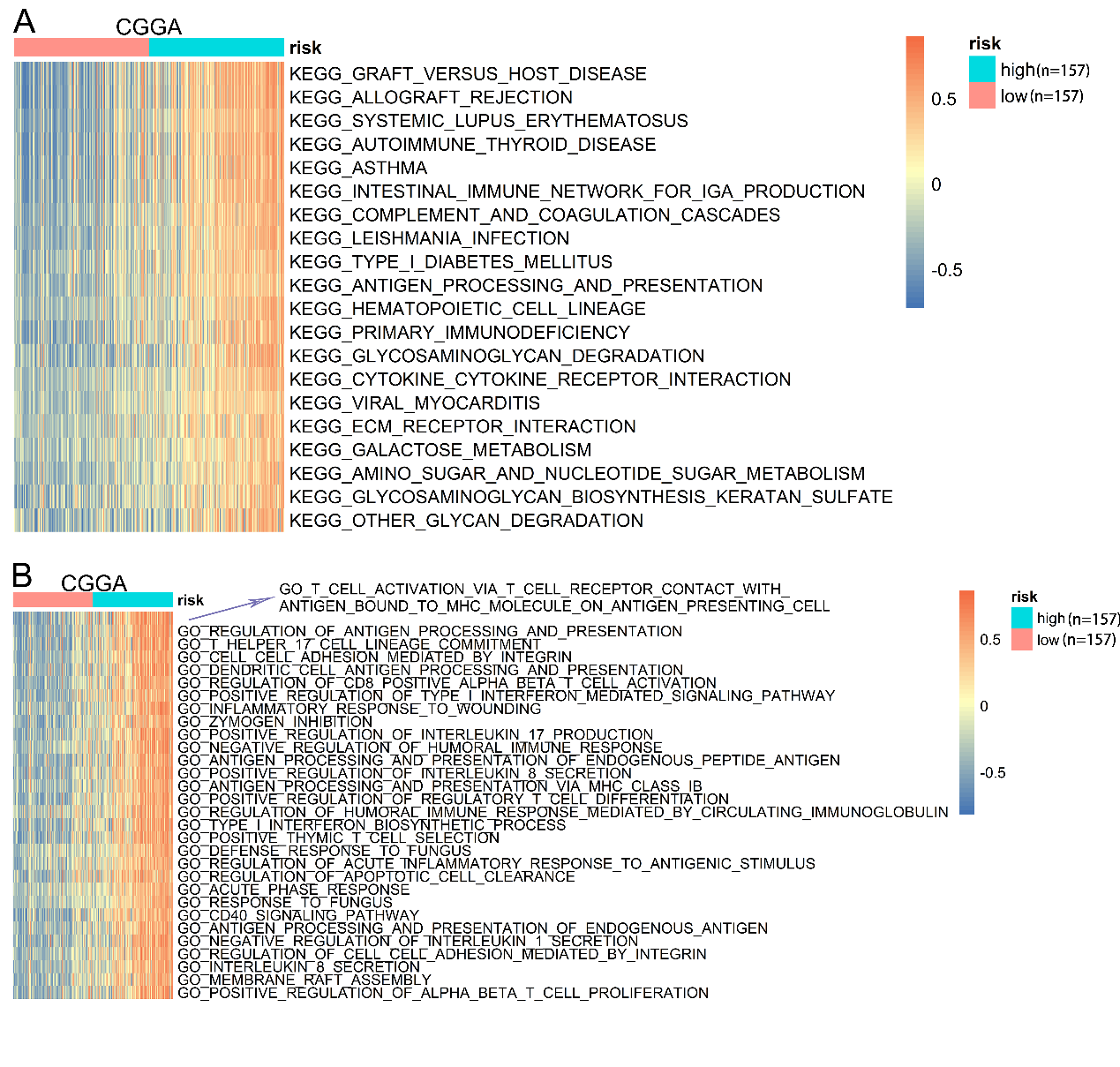


**Supplementary Figure 6.** (A) The top 20 KEGG pathways and (B) the top 30 biological processes enriched in the high-risk group in CGGA.


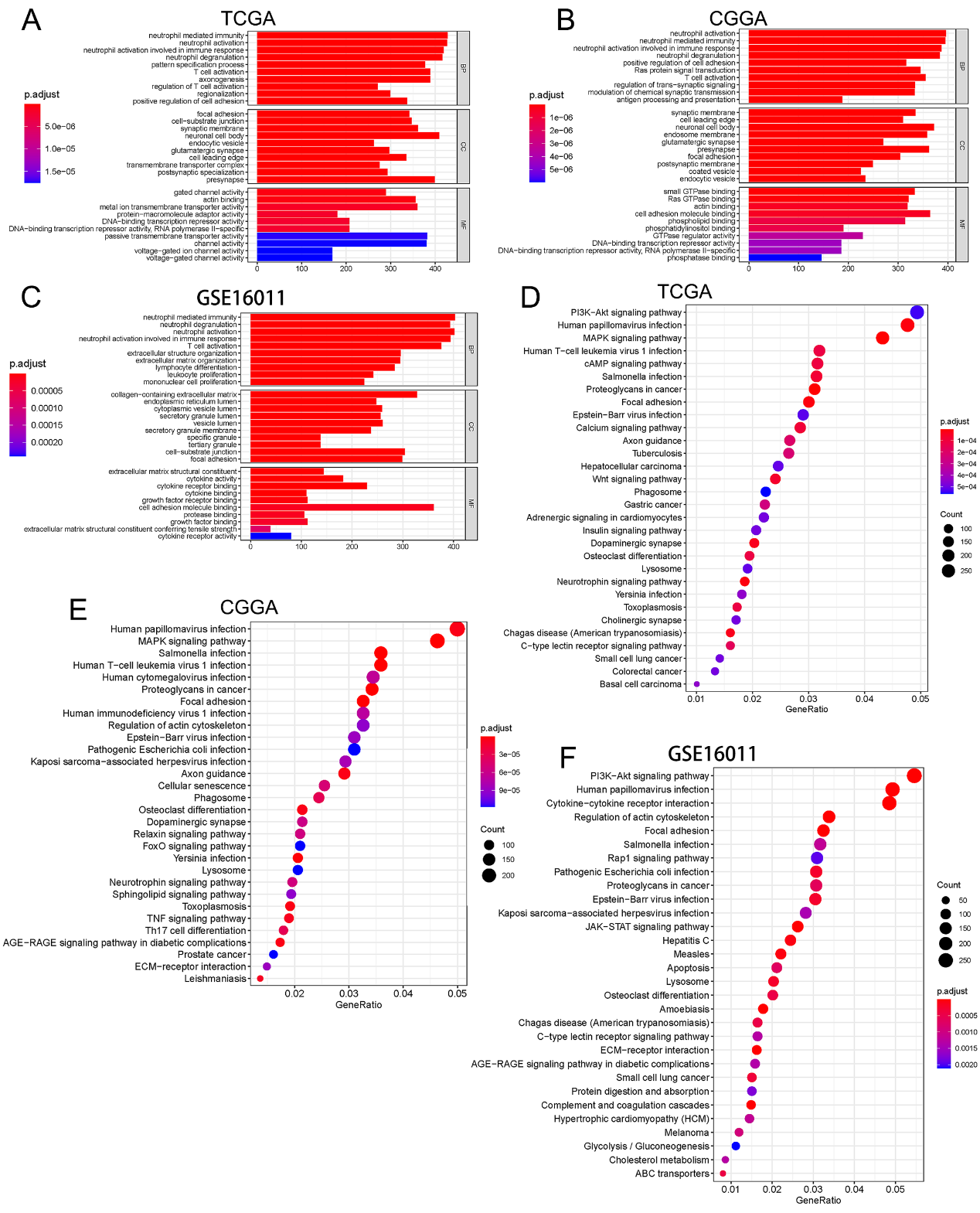
**Supplementary Figure 7.** Functional enrichment analysis based on differentially expressed genes between the high- and low-risk groups. (A−C) GO analysis based on differentially expressed genes between the high- and low-risk groups in (A) TCGA, (B) CGGA, and (C) GSE16011 cohorts. (D−F) KEGG analysis based on differentially expressed genes between the high- and low-risk groups in (D) TCGA, (E) CGGA, and (F) GSE16011 cohorts.


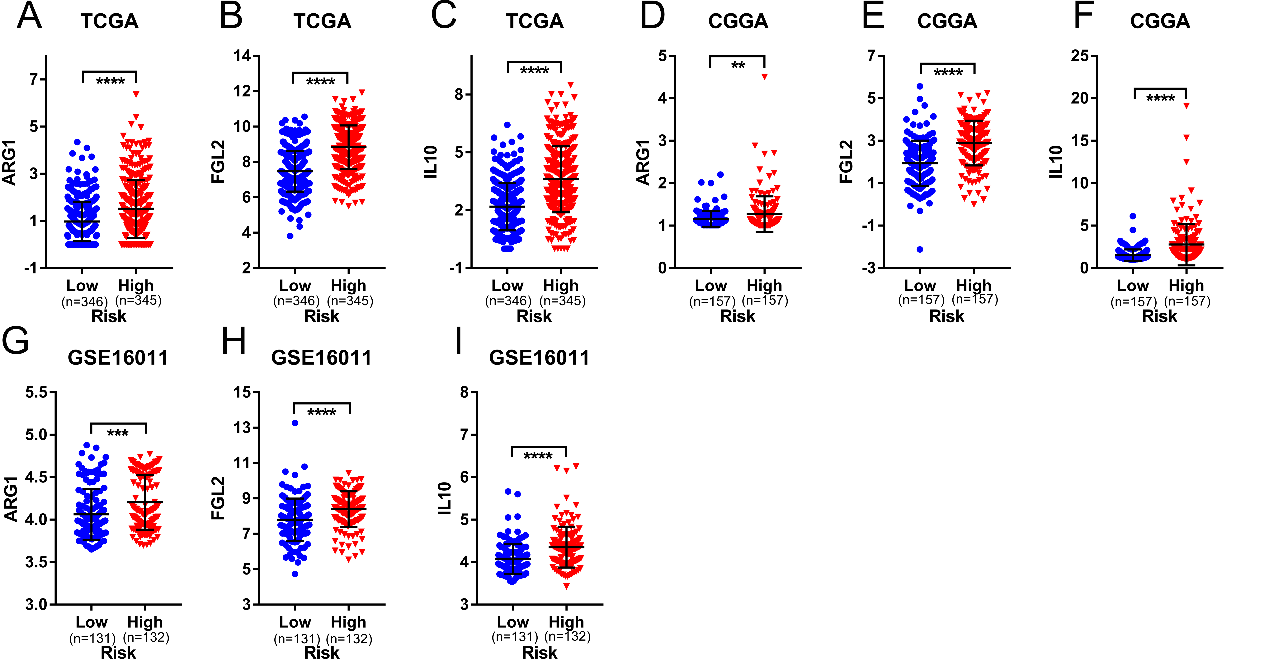
**Supplementary Figure 8.** The expression of ARG1, FGL2, and IL10 in the high- and low-risk groups in (A−C) TCGA, (D-F) CGGA and (G-I) GSE16011 datasets (Wilcoxon test).


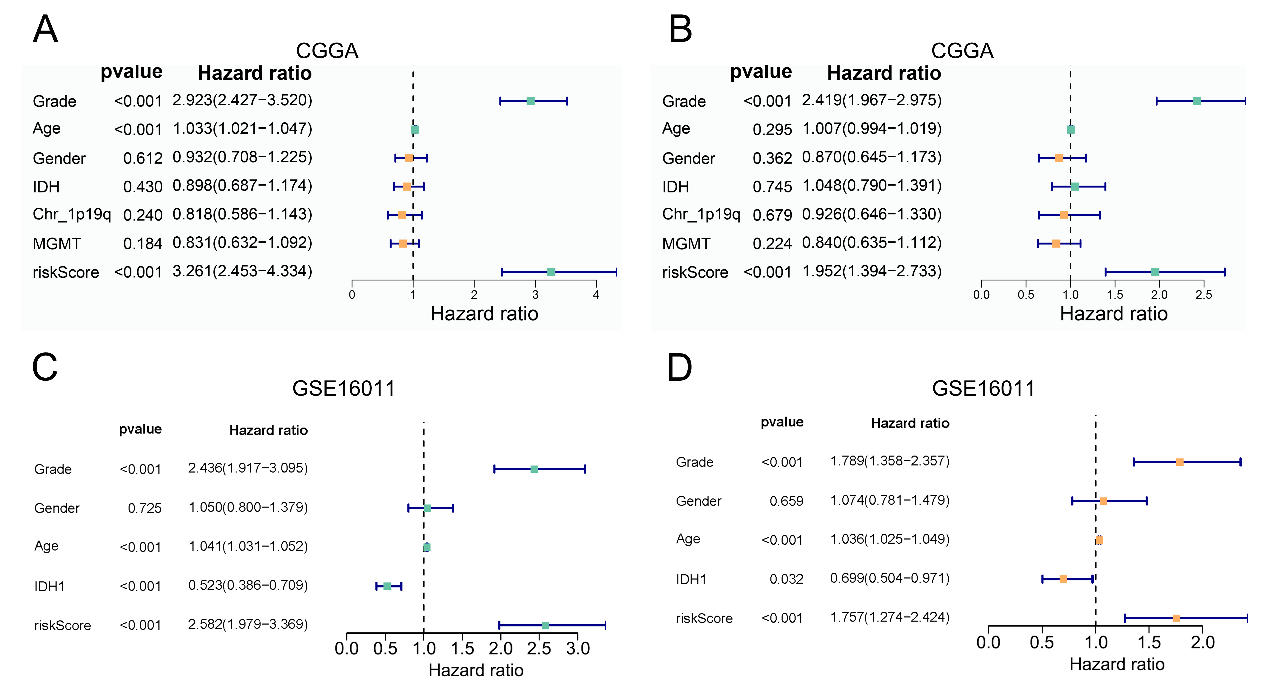
**Supplementary Figure 9.** Forest plot of the univariate (left) and multivariate (right) Cox regression analysis in (A, B) the CGGA and (C, D) the GSE16011 cohorts.
