# Supplementary material for: Characterization of an endoplasmic reticulum stress‐related signature to evaluate immune features and predict prognosis in glioma
Source: J Cell Mol Med. 2021 Feb 21;25(8):3870–84. doi: 10.1111/jcmm.16321 (PMC8051731; doi:10.1111/jcmm.16321)
Supplement: Supplementary file 2 — Table S1 [file JCMM-25-3870-s001.pdf]

Supplementary Table 1: Clinical and molecular characteristics of patients included in this study.

Cohort	TCGA (n=691)	CGGA (mRNAseq_325) (n=314)	CGGA (array) (n=297)	GSE16011 (n=263)
Database	TCGA	CGGA	CGGA	GEO
Platform	Illumina Hiseq 2000 RNAseq	Illumina Hiseq 2000 RNAseq	Agilent Whole Human Genome (Array)	Affymetrix GeneChip Human Genome U133 Plus 2.0 Array
Age(year)				
Mean (range)	46.6(14-89)	43.1(8-79)	42.3(12-70)	50.85(14.38-81.18)
Gender				
Female	294	117	121	87
Male	397	197	176	176
WHO grade				
II	258	100	115	23
III	267	74	56	85
IV	166	140	126	155
TCGA subtype				
Classical	86	72	23	70
Mesenchymal	96	67	108	29
Neural	111	80	80	18
Proneural	238	95	86	38
Unavailable	160	0	0	108
IDH status				

Mutant	437	169	133	80
Wild-type	244	145	162	131
Unavailable	10	0	2	52
1p/19q status				
Codel	168	66	16	0
Non-codel	517	248	74	0
Unavailable	6	0	207	263
MGMT promoter methylation status				
Methylated	489	152	-	0
Unmethylated	165	144	-	0
Unavailable	37	18	-	263

Abbreviations: CGGA, Chinese Glioma Genome Atlas; TCGA, The Cancer Genome Atlas; WHO, World Health Organization; 1p19q Codel, 1p19q codeleted; 1p19qNon-codel, 1p19q non-codeleted.
