# Supplementary material for: Characterization of an endoplasmic reticulum stress‐related signature to evaluate immune features and predict prognosis in glioma
Source: J Cell Mol Med. 2021 Feb 21;25(8):3870–84. doi: 10.1111/jcmm.16321 (PMC8051731; doi:10.1111/jcmm.16321)
Supplement: Supplementary file 3 — Table S2 [file JCMM-25-3870-s002.pdf]

Supplementary Table 2: The clinical characteristics of 12 patients.

Characteristic	Number of patients (n = 12)
Age(years)	
<50	7(58.3%)
≥50	5(41.7%)
Gender	
Male	6(50.0%)
Female	6(50.0%)
WHO grade	
II	4(33.3%)
III	4(33.3%)
IV	4(33.3%)
Primary/Recurrent	
Primary	12(100%)
Recurrent	0(0%)
