# Supplementary material for: Characterization of an endoplasmic reticulum stress‐related signature to evaluate immune features and predict prognosis in glioma
Source: J Cell Mol Med. 2021 Feb 21;25(8):3870–84. doi: 10.1111/jcmm.16321 (PMC8051731; doi:10.1111/jcmm.16321)
Supplement: Supplementary file 4 — Table S3 [file JCMM-25-3870-s003.pdf]

Supplementary Table 3. Primer Sequences for qRT-PCR.

Gene Name	Forward Primer	Reverse Primer
GAPDH	5'-GGAGCGAGATCCCTCCAAAAT-3'	5'-GGCTGTTGTCATACTTCTCATGG-3'
CYP2E1	5'-GTGATGCACGGCTACAAGG-3'	5'-GGGTGGTCAGGGAAAACCG-3'
SLN	5'-ATGGTCCTGGGATTGACTGAG-3'	5'-GTGCCCTCGGATGGAGAATG-3'
BRCA1	5'-GAAACCGTGCCAAAAGACTTC-3'	5'-CCAAGGTTAGAGAGTTGGACAC-3'
CISD2	5'-GTGGCCCGTATCGTGAAGG-3'	5'-CTAGCGAACCCGGTAATGCTT-3'
LRRK2	5'-GAGCACGCCTCCAAGTTATTT-3'	5'-ACTGGCATTATGAACTGTTAGCA-3'
BMP2	5'-ACCCGCTGTCTTCTAGCGT-3'	5'-TTTCAGGCCGAACATGCTGAG-3'
MYH7	5'-ACTGCCGAGACCGAGTATG-3'	5'-GCGATCCTTGAGGTTGTAGAGC-3'
HSPB1	5'-ACGGTCAAGACCAAGGATGG-3'	5'-AGCGTGTATTTCCGCGTGA-3'
DNM1L	5'-CTGCCTCAAATCGTCGTAGTG-3'	5'-GAGGTCTCCGGGTGACAATTC-3'
SHISA5	5'-ACCTGTGATGACCAATACTGCT-3'	5'-TCCACCGGCTCTACACTGG-3'
RNF185	5'-GTGTTTACATCAGTGGTTGGAGA-3'	5'-GTGCTGCCCCTTCCATAGAG-3'
RCN1	5'-AAACGGGTGCAGAAAAGATACA-3'	5'-AGGTAGTAACCATAGGTGGCTT-3'
SPP1	5'-CTCCATTGACTCGAACGACTC-3'	5'-CAGGTCTGCGAAACTTCTTAGAT-3'
RPN2	5'-TGGCCCTGACAATCATAGCC-3'	5'-GAGTCCCACGATGGAGTAGAA-3'
PDIA3	5'-GCCTCCGACGTGCTAGAAC-3'	5'-GCGAAGAACTCGACGAGCAT-3'
ATP2A2	5'-CATCAAGCACACTGATCCCGT-3'	5'-CCACTCCCATAGCTTTCCCAG-3'
